# Oxytocin Reverses Osteoporosis in a Sex-Dependent Manner

**DOI:** 10.3389/fendo.2015.00081

**Published:** 2015-05-19

**Authors:** Guillaume E. Beranger, Mansour Djedaini, Séverine Battaglia, Christian H. Roux, Marcel Scheideler, Dominique Heymann, Ez-Zoubir Amri, Didier F. Pisani

**Affiliations:** ^1^UMR 7277, Institut of Biology Valrose (iBV), Université de Nice-Sophia-Antipolis, Nice, France; ^2^UMR 7277, Institut of Biology Valrose (iBV), CNRS, Nice, France; ^3^U1091, INSERM, Institut of Biology Valrose (iBV), Nice, France; ^4^Université de Nantes, Nantes, France; ^5^UMR 957, INSERM, Equipe LIGUE Nationale Contre le Cancer 2012, Nantes, France; ^6^Service de Rhumatologie, Hopital l’Archet CHU, Nice, France; ^7^Helmholtz Center Munich, Institute for Diabetes and Cancer, Neuherberg, Germany; ^8^Heidelberg University Hospital, Heidelberg, Germany; ^9^German Center for Diabetes Research (DZD), Neuherberg, Germany

**Keywords:** orchidectomy, bone, adipose tissue, male, female, mice

## Abstract

The increase of life expectancy has led to the increase of age-related diseases such as osteoporosis. Osteoporosis is characterized by bone weakening promoting the occurrence of fractures with defective bone regeneration. Men aged over 50 have a prevalence for osteoporosis of 20%, which is related to a decline in sex hormones occurring during andropause or surgical orchidectomy. As we previously demonstrated in a mouse model for menopause in women that treatment with the neurohypophyseal peptide hormone oxytocin (OT) normalizes body weight and prevents the development of osteoporosis, herein we addressed the effects of OT in male osteoporosis. Thus, we treated orchidectomized mice, an animal model suitable for the study of male osteoporosis, for 8 weeks with OT and then analyzed trabecular and cortical bone parameters as well as fat mass using micro-computed tomography. Orchidectomized mice displayed severe bone loss, muscle atrophy accompanied by fat mass gain as expected in andropause. Interestingly, OT treatment in male mice normalized fat mass as it did in female mice. However, although OT treatment led to a normalization of bone parameters in ovariectomized mice, this did not happen in orchidectomized mice. Moreover, loss of muscle mass was not reversed in orchidectomized mice upon OT treatment. All of these observations indicate that OT acts on fat physiology in both sexes, but in a sex specific manner with regard to bone physiology.

## Introduction

Life expectancy continually increases in industrialized countries and is linked to aging-associated phenotypes such as a decrease in hormonal levels, lean and bone mass, as well as gain in fat mass. Along with aging-associated diseases defined as a major cause of morbidity and mortality, osteoporosis can be found essentially associated to a severe decrease in circulating estrogens due to menopause in women (1). Although with lower frequency than in women, the osteoporosis in men aged over 50 has a prevalence of 20% and is a relatively common burden with treatments less defined than in women (2).

Osteoporosis, defined by the deterioration of bone density and microarchitecture leading to bone fragility (3), is a major public health problem with 6.3 million of cases expected in 2050 (4). Besides substantial financial costs, this disease leads to a deleterious life quality with a significant decrease in mobility up to the loss of autonomy, a susceptibility to other diseases such as osteoarthritis and obesity, and finally a significant increase in morbidity and mortality, especially in the course of the year following a hip fracture. Osteoporosis in men is less well known, but seems to be also associated with a decrease in sex hormone levels (androgens), particularly testosterone, and is aggravated by environmental factors such as nutrition and physical inactivity (2). It is difficult to anticipate the onset of osteoporosis in men (5), except after surgical removal of the testis (orchidectomy), for example, in case of prostate cancer treatment to reduce testosterone level, which leads to a drop in the level of circulating androgens (6). In these cases, preventive postoperative treatments can be provided to limit the development of osteoporosis. As for women, a decrease of sex hormones can also cause weight gain, behavioral changes, and muscle wasting in men (5).

Osteoporosis may affect trabecular or cortical bone associated with different types of fractures (7, 8). The strength and integrity of the bone depend on the balance between resorption by osteoclasts and bone formation by osteoblasts (9, 10). The decrease of bone mass, which occurs during osteoporosis, implies an acceleration of the “bone turnover” with an imbalance between resorption and formation in favor of bone resorption (11, 12).

In addition to bone loss, osteoporosis is associated with a gain in bone marrow adiposity due to the formation of adipocytes, at the expense of osteoblasts, both derived from a common progenitor: the mesenchymal stem cell. Adiposity is also connected to the prevalence of bone fracture in osteoporosis (13). The adipocyte/osteoblast balance is essential for bone homeostasis, as it determines (1) the amount of essential osteoblasts for bone formation and (2) the secretion of adipokines involved in bone metabolism. We previously characterized the major role of this balance in the pathophysiology of osteoporosis, which is controlled by a circulating hormone, namely oxytocin (OT). *In vitro*, the treatment of mesenchymal stem cells with O promotes the formation of osteoblasts and inhibits adipogenesis (14). In addition, we showed an inverse correlation between OT circulating levels and the occurrence of osteoporosis (14–16) on a small cohort of post-menopausal women first, and then on a larger cohort from a European prospective multicenter study (OPUS). Finally, we demonstrated that treatment of ovariectomized mice, an animal model mimicking menopause in women, by daily injections of OT (1 mg/kg/day) prevented and reversed the osteoporotic phenotype. Indeed, both OT preventive and curative treatments led to normalization of bone parameters (morphometric and mechanical), to a decrease of bone marrow adiposity and, interestingly, to a normalization in weight gain induced by ovariectomy (17).

Oxytocin belongs to the family of pituitary hormones and regulates the function of peripheral organs including the mammary glands and smooth muscle of the uterus. Due to this property, it has commonly been used in other medical obstetrics without significant side effects (18). Thus, OT emerges as a promising molecule in the treatment of osteoporosis.

Herein, we daily injected orchidectomized male mice with 1 mg/kg of OT for 8 weeks to analyze its effect on male osteoporosis. We previously used this protocol with success to reverse the bone phenotype in ovariectomized female mice (17, 19). Analysis of treated mice demonstrated that OT did not restore the bone loss and muscle atrophy in orchidectomized male mice. These results demonstrated a sex-dependent efficiency of OT on bone remodeling following gonadectomy that might be linked to the difference in sexual hormones between male and female.

## Materials and Methods

### Animals

The experiments were conducted in accordance with the French and European regulations for the care and use of research animals and were approved by the local experimentation committee. Animals were maintained under constant temperature (21 ± 2°C) and 12:12-hour light–dark cycles, with *ad libitum* access to standard chow diet and water. Ten-week-old C57Bl/6J mice were randomized and subjected to bilateral orchidectomy, ovariectomy or sham surgery by the manufacturer (Charles River Laboratories). For sham-operated mice, the testis or ovaries were exteriorized but replaced intact. Two weeks after surgery, orchidectomized (ORX), ovariectomized (OVX), or sham groups of mice (*n* = 12) were intra-peritoneally injected with vehicle (Ve, NaCl 0.9%) or OT (1 mg/kg) on a daily basis and over 8 weeks.

### Micro-computed tomography

Trabecular bone microarchitecture of the distal femoral metaphysis was analyzed using the high-resolution SkyScan-1076 X-ray micro-computed tomography system (SkyScan). Femora were scanned after necropsy using the same parameters: 9 μm of pixel size, 49 kV, 0.5 mm thick aluminum filter, and 0.5° of rotation step. Calculation of femur trabecular bone parameters (Bone Volume/Total Volume: BV/TV) following 3D morphometric parameters (Bone ASBMR nomenclature) were performed on 0.9 mm of femur length from the distal growth plate excluding the cortical bone. Analysis of cortical bone was performed using the same parameters. Cortical cross-sectional area (CSA) was determined on a transversal section localized at the mid-length of the femoral shaft.

Adipose tissue quantification was carried out using a SkyScan-1178 X-ray micro-computed tomography system. Mice were anesthetized and scanned using the same parameters: 104 μm of pixel size, 49 kV, 0.5 mm thick aluminum filter, and 0.9° of rotation step. Total adipose tissue volume was determined between the lumbar vertebra 1 (L1) and the caudal vertebra 4 (C4), whereas intra-abdominal and subcutaneous adipose tissues areas were measured on one section at the lumbar 5 (L5) level. 3D reconstructions and analysis of bone parameters and adipose tissue areas or volumes were performed using NRecon and CTAn software (SkyScan).

### Plasma measurements

Blood was sampled by intracardiac puncture from anesthetized animals and plasma was stored at −80°C. Procollagen I N-Terminal propeptide (PINP, USCN) or Cross Linked C-Telopeptide of Type I Collagen (CTXI, USCN) plasma levels was measured using an ELISA kit as per the manufacturer’s instructions.

### Isolation and analysis of RNA

Total RNA was extracted using a TRI-Reagent kit as per the manufacturer’s instructions. For total bone RNA extraction, humeri were homogenized using an Ultra Turrax homogenizer and samples were then processed as per the TRI-Reagent extraction protocol. Two micrograms of total RNA, digested with DNAse I (Promega), were subjected to reverse transcription-polymerase chain reaction (RT-PCR) analysis as previously described (20). Quantitative PCR assays were run using an ABI Prism 7,000 real-time PCR machine (PerkinElmer Life and Analytical Sciences). The final reaction volume was 20 μl, including 100 nM of specific primers, 20 ng of reverse-transcribed RNA, and 10 μl of SYBR Green master mix (Applied Biosystems). Quantitative PCR conditions were as follows: 2 min at 50°C; 10 min at 95°C; and 40 cycles of 15 s at 95°C, 1 min at 60°C. The expression of genes was normalized to the expression of two housekeeping genes, 36B4 and hprt. Gene expression was quantified using the comparative −ΔCt method. The oligonucleotides for each target of interest, designed using Primer Express software (PerkinElmer Life and Analytical Sciences), are shown (forward and reverse) in Table 1.

**Table 1:** **Sequence of primers used for gene expression analysis**.

Target gene	Forward	Reverse
*RANKL*	GGAACTGCAACACATTGTGGG	GCCTTCCATCATAGCTGGAGC
*OPG*	TCCGGCGTGGTGCAA	AGAACCCATCTGGACATTTTTTG
*Tracp*	TGCCTACCTGTGTGGACATGA	CACATAGCCCACACCGTTCTC
*Collagen 1 alpha 1*	GCGAAGGCAACAGTCGCT	CTTGGTGGTTTTGTATTCGATGAC
*Osteocalcin*	CCACCCGGGAGCAGTGT	CTAAATAGTGATACCGTAGATGCGTTTG
*Hprt*	GCCTAAGATGAGCGCAAGTTGA	AGGCAGATGGCCACAGGACTA
*36B4*	TCCAGGCTTTGGGCATCA	CTTTATCAGCTGCACATCACTCAGA

### Statistical analyses

Data are expressed as mean values ±SEM and was analyzed using the two-tailed Student’s *t*-test. Differences were considered statistically significant at *p* ≤ 0.05.

## Results

### Oxytocin treatment does not restore bone parameters in orchidectomized mice

**Figure 1:**
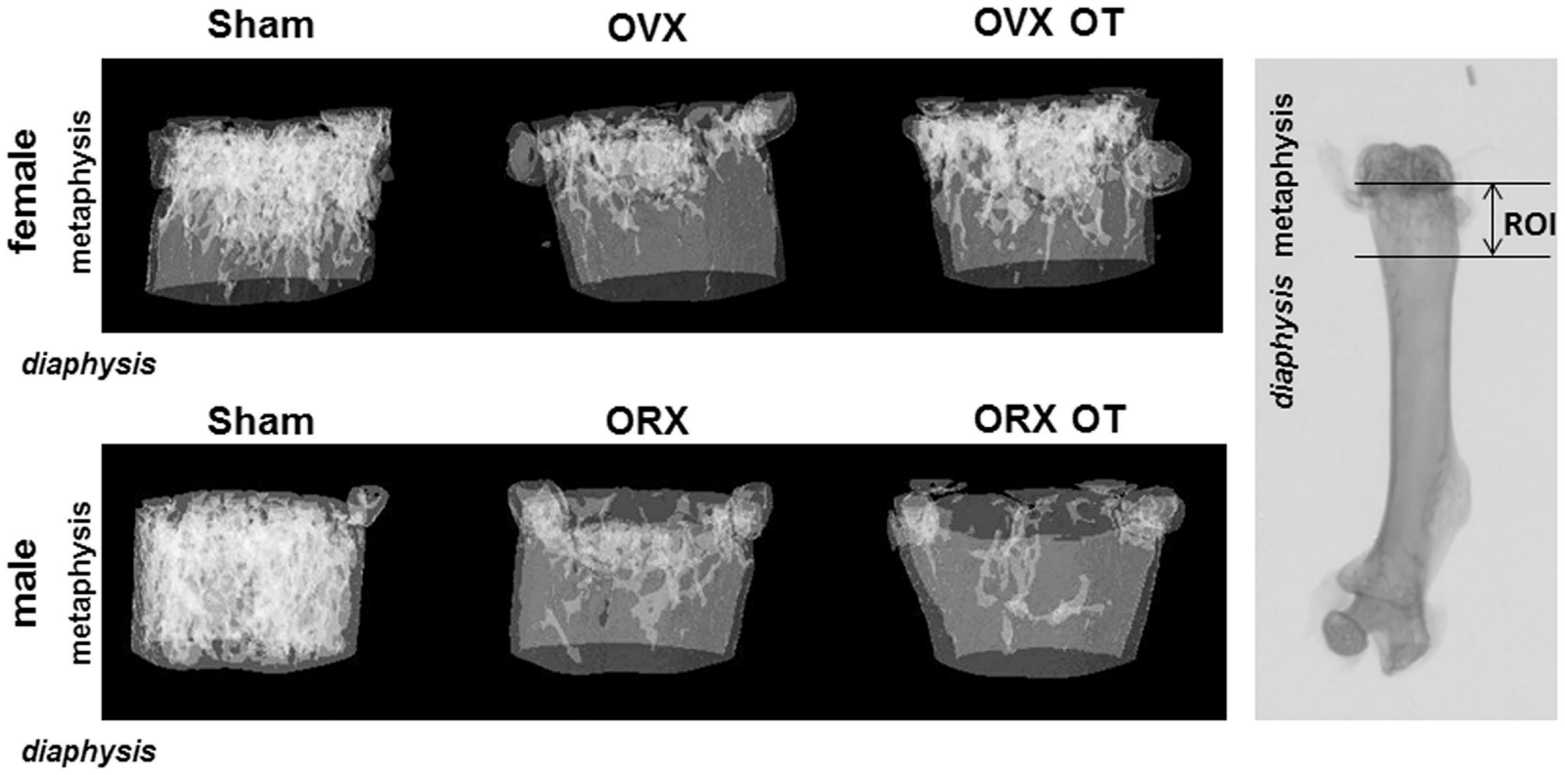
**Micro-computed tomography of distal femur metaphysis of OVX and ORX mice injected or not with OT and their corresponding Sham mice**. A three-dimensional representation of a horizontal analysis of femurs from a representative mouse of each group is shown. Right picture displayed a femur radiography with the region of interest (ROI) used for 3D reconstruction.

**Figure 2:**
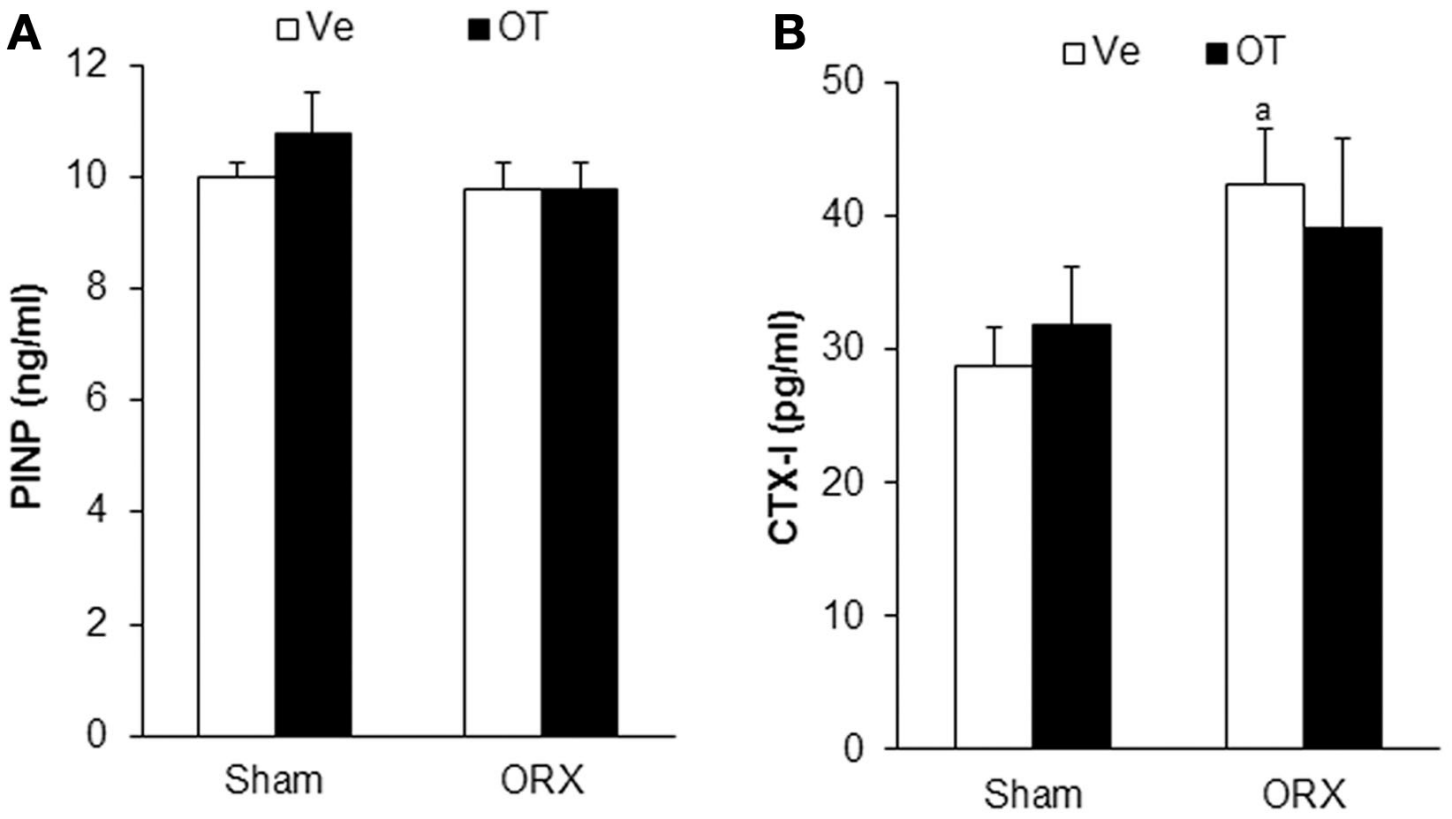
**Plasmatic bone remodeling markers**. Plasma levels of bone formation marker PINP **(A)** and bone resorption marker CTXI **(B)** in Sham and ORX mice following 8 weeks of OT or Ve treatment. Data are represented as mean ± SEM. **(A)** *p* < 0.05 vs. Sham-Ve (*n* = 12 mice/group).

We analyzed bone parameters upon OT treatment in orchidectomized (ORX) and ovariectomized (OVX) mice, animal models for male and female osteoporosis, respectively. Two weeks post-surgery, controls (male or female Sham) and ORX or OVX mice were daily injected with OT at a 1 mg/kg dose or vehicle (Ve, NaCl 0.9%). After 8 weeks of treatment, trabecular and cortical bone parameters were analyzed using high-resolution micro-computed tomography (MicroCT). We confirmed our previous observations showing that OT treatment restored bone architecture of OVX mice (14, 17). As expected in ORX mice, orchidectomy induced a dramatic trabecular bone loss evidenced by the significant decline in BV/TV, trabecule thickness (Tb. Th.), and trabecule number (Tb. N.) as well as by an increase in the trabecule spacing (Tb. Sp.) compared to the Sham group (Table 2). In a similar way, cortical bone was also affected upon orchidectomy as depicted by the significant decrease of mid-diaphysis cortical CSA as shown in Table 2. In contrast to OVX mice, none of both cortical and trabecular parameters were restored in ORX mice upon OT treatment for 8 weeks as observed in Figure 1. In parallel, we quantified various bone remodeling markers in plasma from Sham and ORX mice treated with OT or vehicle. Regarding bone formation, neither orchidectomy nor OT injection modified its activity according to the plasma level of PINP (Figure 2A). In terms of bone resorption, we measured a significant increase in the level of CTXI in ORX mice, which was not significantly modified upon OT treatment (Figure 2B).

**Table 2:** **MicroCT analysis of femora trabecular and cortical bone parameters**.

	Mice group	Sham-Ve (*n* = 12)	Sham OT 1 mg/kg (*n* = 12)	ORX Ve (*n* = 12)	ORX OT 1 mg/kg (*n* = 12)
**Trabecular bone**					
Distal femoral metaphysis	BV/TV (%)	15.4 ± 1.2	11.9 ± 1.2	3.2 ± 0.3^a^	3.1 ± 0.3^a^
	Tb. Th. (μm)	69 ± 1	63 ± 2^a^	65 ± 1^a^	62 ± 1^a^
	Tb. N. (mm^−1^)	2.2 ± 0.1	1.9 ± 0.1	0.5 ± 0.04^a^	0.5 ± 0.04^a^
	Tb. Sp. (μm)	238 ± 9	247 ± 6	532 ± 23^a^	528 ± 16^a^
**Cortical bone**					
Mid-femur diaphysis	CSA (mm^2^)	1.04 ± 0.08	0.97 ± 0.06^a^	0.83 ± 0.04^a^	0.83 ± 0.04^a^

Control (Sham) and ORX mice were submitted to daily injections of OT or Ve for 8 weeks starting 2 weeks after surgery. Detailed analysis of trabecular parameters was performed on the distal metaphysis of femora and on the mid-femur diaphysis for cortical bone parameters. Bone volume/total volume (BV/TV), trabecular thickness (Tb. Th), trabecular spacing (Tb. Sp), trabecular number (Tb. N), and cortical cross-sectional area (CSA) are reported in the Table 1.

^a^*p* < 0.05 vs. Sham-Ve (*n* = 12 mice/group). Data are represented as mean ± SEM.

### OT treatment does not significantly impact the expression of bone molecular markers *In vivo*

**Figure 3:**
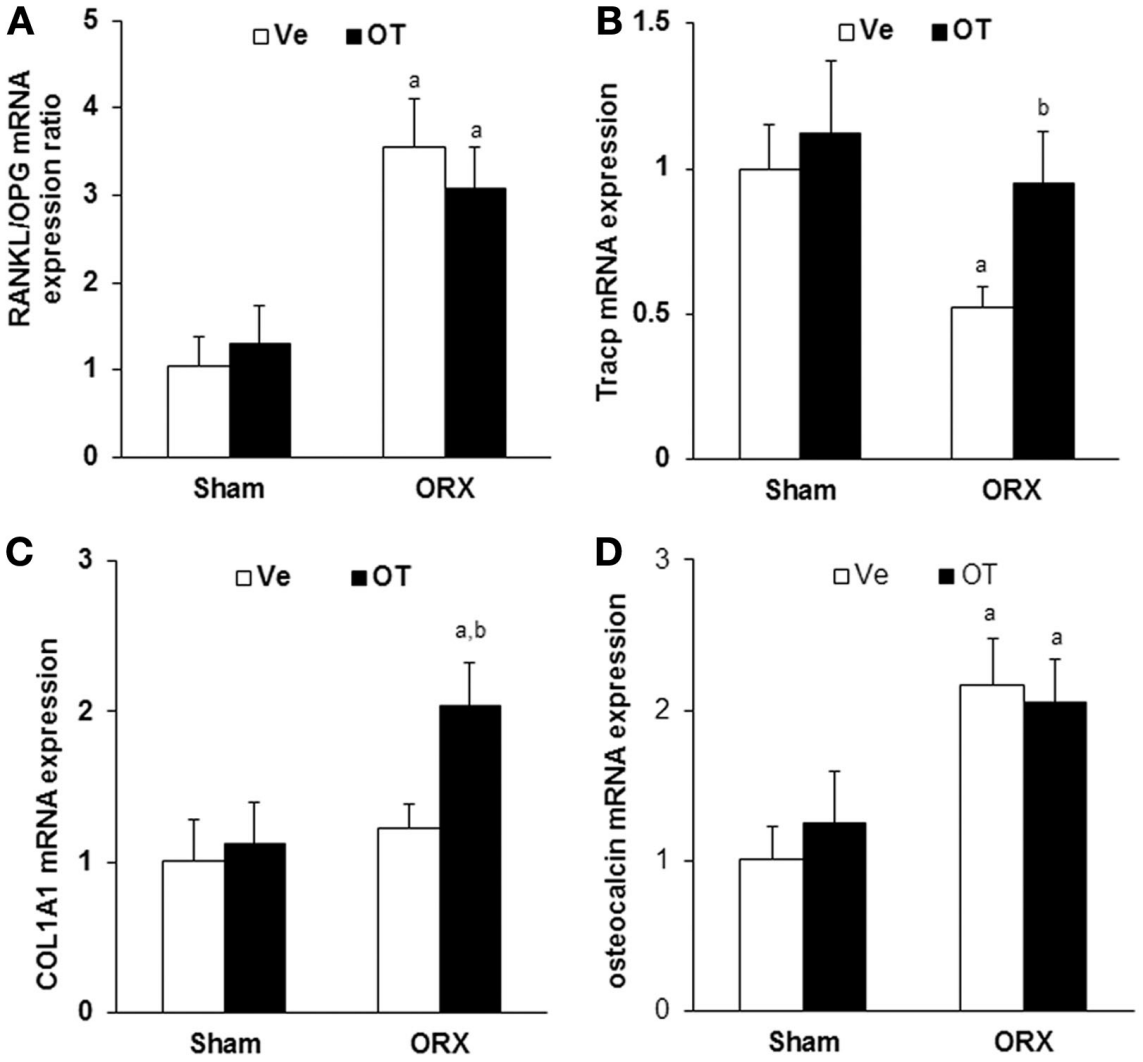
***In vivo* analysis of bone molecular markers**. Real-Time PCR for **(A)** RANKL, osteoprotegerin (OPG), **(B)** Tracp, **(C)** collagen 1 alpha 1 (COL1A1), and **(D)** osteocalcin expression on RNA from humeri of Sham and ORX mice submitted to daily injections of OT or Ve for 8 weeks starting 2 weeks after surgery (*n* = 12 mice/group). Data are represented as mean ± SEM. **(A)** *p* < 0.05 vs. Sham-Ve and **(B)** *p* < 0.05 vs. ORX-Ve.

Next, we analyzed the expression of bone remodeling markers *in vivo* to address the effect of OT at the molecular level. We quantified mRNA expression using real-time PCR and mRNAs isolated from total long bones of Sham and ORX mice treated with vehicle or OT. We observed that orchidectomy induced a threefold increase of RANKL/OPG mRNA ratio, indicating an increase of pro-osteoclastogenic signaling. RANKL/OPG ratio was not affected by OT treatment and was maintained at a high level compared to the Sham group (Figure 3A). Regarding bone resorption, mRNA expression of the osteoclastogenic marker Tracp decreased upon orchidectomy and was normalized after OT treatment (Figure 3B). As expected, similar observations were obtained for OVX mice [data not shown (17)]. We measured the expression of osteoblast markers such as collagen 1 alpha 1 (COL1A1) and osteocalcin. COL1A1 mRNA expression was not affected by orchidectomy but was induced by OT treatment in the ORX group (Figure 3C). Osteocalcin expression was induced upon orchidectomy and was not restored in ORX-OT mice group (Figure 3D). Opposite data were obtained for female mice, a decrease in COL1A and osteocalcin mRNA upon ovariectomy that were restored upon OT treatment [data not shown (17)].

### Oxytocin treatment does not normalize orchidectomy-induced body weight loss

**Figure 4:**
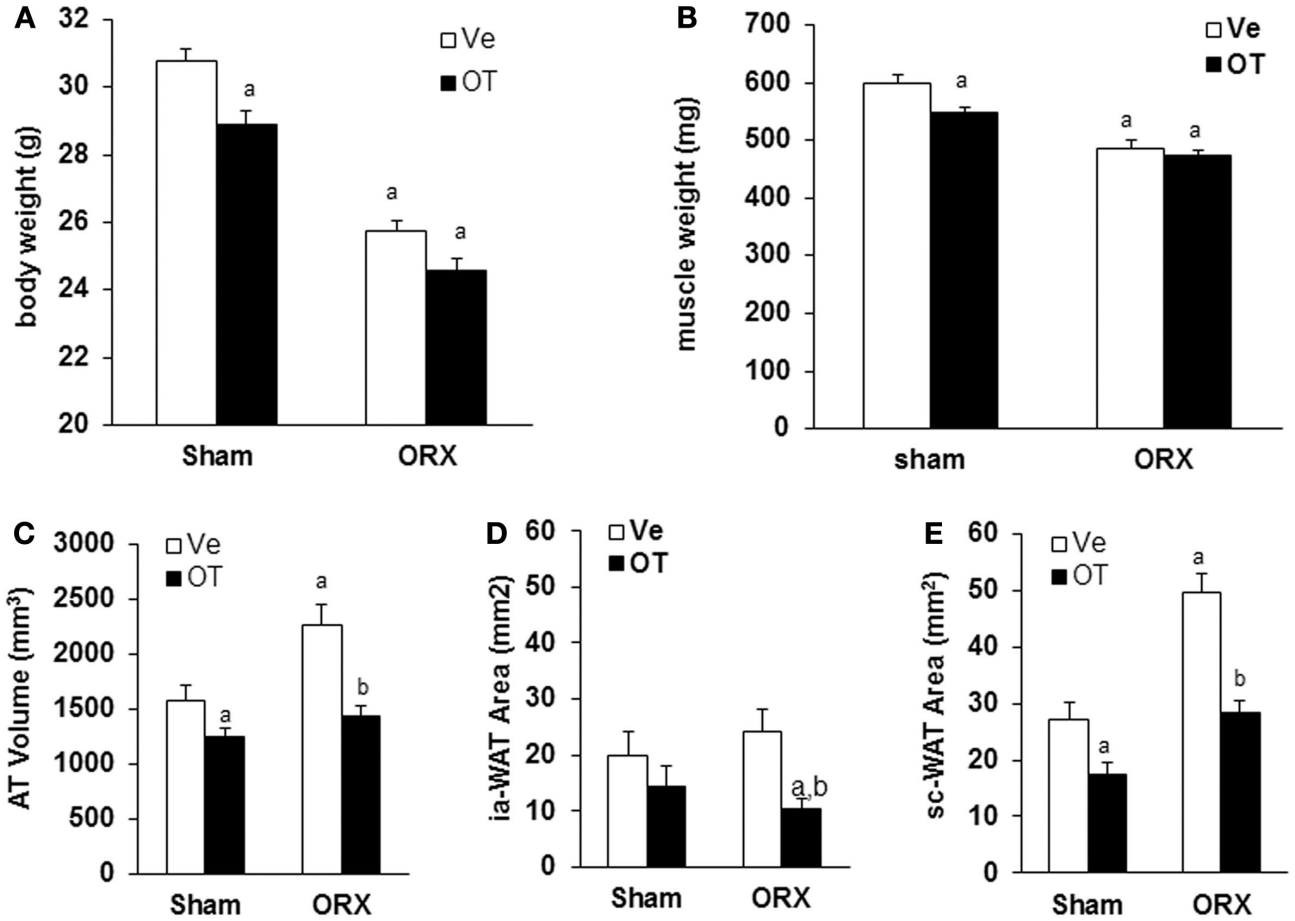
**Effects of OT on body weight, muscle weight, and fat mass in ORX mice**. Sham and ORX mice were submitted to daily injections of OT or Ve for 8 weeks starting 2 weeks after surgery. **(A)** Body and **(B)** muscle weights were measured at the end of treatment. **(C)** The volume of white adipose tissue between lumbar vertebra 1 (L1) and the caudal vertebra 4 (C4) was measured using micro-computed tomography. **(D,E)** Intra-abdominal and subcutaneous adipose tissue areas were measured on one section at the L4/L5 (lumbar vertebra 4 and 5) junction level (*n* = 12 mice/group). Data are represented as mean ± SEM. **(A)** *p* < 0.05 vs. Sham-Ve and **(B)** *p* < 0.05 vs. ORX-Ve.

Then, we analyzed body weight and body composition in mice upon OT or Ve treatment for 8 weeks. We previously observed that, in OVX mice displaying an increase of both body weight and fat mass, OT was able to restore both parameters to levels of the control Sham group [data not shown (17)]. As previously described (21), ORX mice displayed a dramatic loss of total body weight (≈5 g) mainly due to a decrease in lean mass, especially in muscle mass, in the absence of testosterone (Figure 4). However, daily OT injections for 8 weeks did not restore body weight and muscle mass compared to Sham-Ve mice (Figure 4).

### Oxytocin treatment normalizes the increase of orchidectomy-induced fat mass

**Figure 5:**
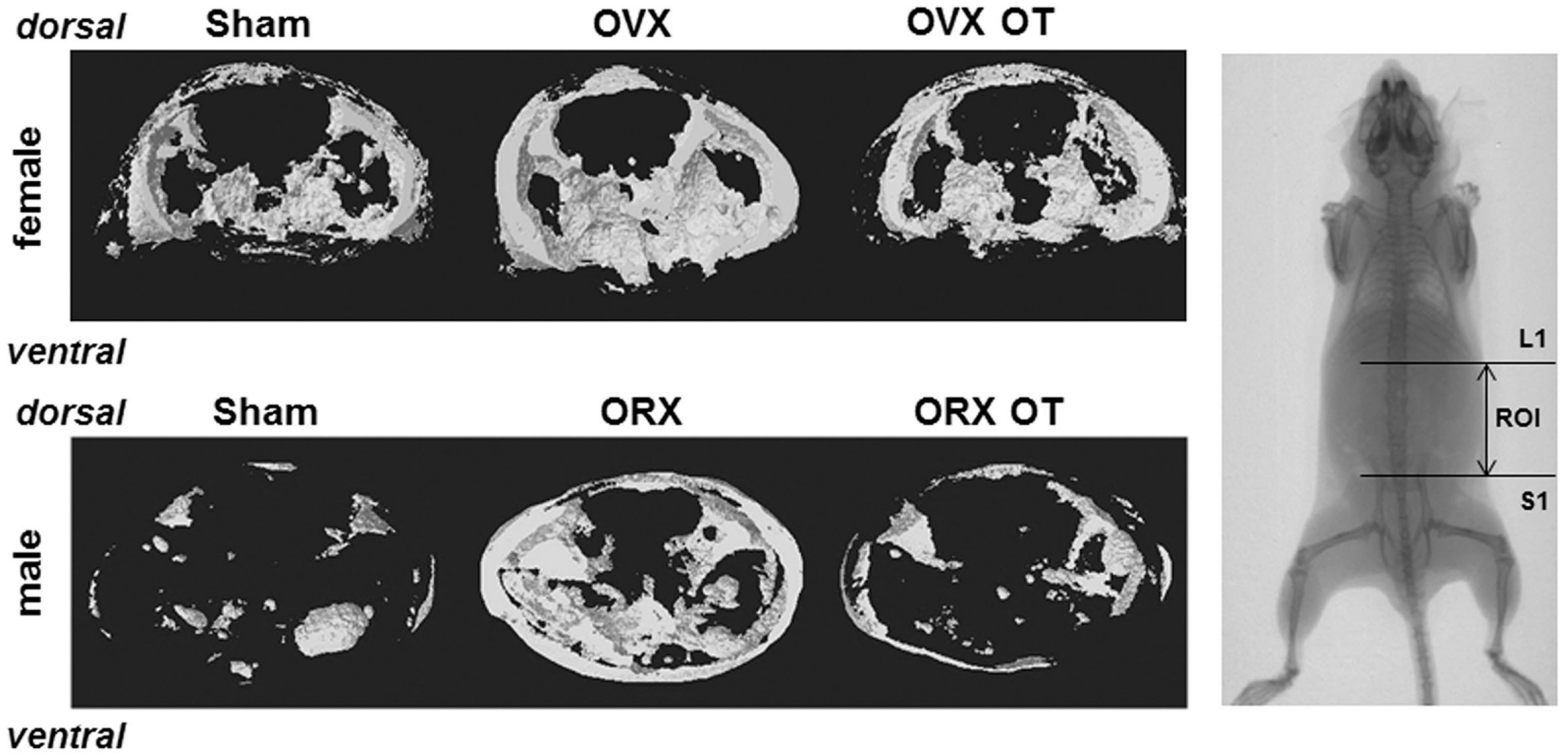
**Micro-computed tomography analysis of abdominal fat of OVX and ORX mice injected or not with OT and their corresponding Sham mice**. A three-dimensional representation of abdominal fat between lumbar vertebra 1 (L1) and sacral 1 (S1) from a representative mouse of each group is shown. Right pictures displayed a whole mouse radiography with the ROI (region of interest) used for 3D reconstruction.

As we showed that the OT treatment reversed ovariectomy-induced fat mass gain, we aimed to know whether this is also the case for male mice. Using micro-computed tomography, we quantified adipose tissue (AT) volume between the lumbar vertebra 1 (L1) and the caudal vertebra 4 (C4). We found a 50% increase in fat volume within this area in ORX compared to Sham mice (Figure 4C). Interestingly, we observed that OT restored the adipose tissue volume in ORX mice to the level of that observed in Sham-Ve mice, and a significant decrease of AT volume in Sham-OT treated mice was also observed. We next quantified the intra-abdominal and subcutaneous white adipose tissue (WAT) by measuring their respective areas on a MicroCT section at the L4/L5 junction. We observed that orchidectomy induced a significant increase of the subcutaneous WAT depot compared to the intra-abdominal depot (Figures 4D,E). Finally, OT treatment decreased both depots and more specifically restored the surface of subcutaneous WAT to the level observed in Sham-Ve mice. Thus, we observed that OT rescues the increase of the orchidectomy-induced WAT depot in a similar manner as that observed in female OVX mice (Figure 5).

## Discussion

As for post-menopausal women or female animal models of osteoporosis (ovariectomized mice, OVX), severe bone loss occurs in men suffering from testicular dysfunction or in the corresponding male animal models (orchidectomized mice, ORX). Osteoporosis in hypogonadal men is characterized by an elevated bone turnover, resulting in a sustained loss of bone mass (22), which is paralleled by decreased muscle mass and increased fat mass (21, 23). Orchidectomy in male rodents induces trabecular and cortical bone loss, a lowered muscle mass – assessed by lean body mass – with a concomitant gain in fat mass (24, 25). Furthermore, as observed for hypogonadal men (21), testosterone treatment prevents bone loss and body composition changes in these rodents (25).

We and others demonstrated that OT plays a key role in the physiological regulation of bone, muscle, and adipose tissue (14, 17, 19, 26). Of note, it has been established that OT pathway was involved in the physiology of bone remodeling for both genders by the analysis of OT and OTR knock-out mice (27). OT and OTR knock-out mice display an osteoporotic phenotype without any sex difference according to the authors. Using OVX mice as an animal model mimicking post-menopausal osteoporosis, we demonstrated that OT prevented and reversed osteoporosis in mice and also normalized body weight. The later effect was mainly due to a decrease in fat mass (14, 17). In this study, we addressed the role of OT on bone tissue and body composition using ORX mice, *in fine* to investigate if OT could be considered as a therapeutic treatment option for male osteoporosis at the expense of testosterone.

Our study demonstrates that daily injection of OT in ORX mice did not restore the osteoporotic phenotype. We assume that this difference regarding the regulation of bone remodeling and homeostasis upon gonadectomy in male and female, could be due to differences in the physiopathological process of osteoporosis induced either by testis or ovary removal. Actually, if the phenotypical outcome remains the same for both genders, it is known that the decline of the hormonal level displays some common and some different features depending if it occurs in male or female. On one hand, it is known that one of the first consequences of gonads removal is an increase of the osteoclastogenic potential of bone marrow cells leading to an increase of bone resorption (28). But on the other hand, if the periosteal bone formation rate is increased in OVX rats, there is a decrease in this periosteal bone formation rate in ORX rats (29).

Estrogens (i.e., estradiol) and androgens (i.e., testosterone) are sex steroid hormones displayed by the two genders, but with different levels, i.e., higher testosterone levels found in male and higher estradiol levels found in female. A proportion of estradiol originates from testosterone through its conversion by cytochrome p450 aromatase (30). Moreover, estradiol as well as testosterone treatments restored bone loss in case of hypogonadism by suppressing bone resorption (31). Despite these common features, estradiol and testosterone do not regulate bone remodeling through the same cellular pathways. It has been demonstrated that testosterone was able to regulate bone formation and resorption activity through both direct and indirect inhibitory effects on osteoblasts and osteoclasts. By contrast, estradiol targets only osteoclast formation using a non-direct pathway through osteoblastic cells (32). We observed in our previous work that OT regulates bone remodeling in OVX mice through an effect on osteoblasts (14, 17). This observation might explain that OT can replace estrogens in OVX mice and rescue the osteoporotic phenotype but not in ORX mice for which androgens are missing. However, estrogens are able to rescue the osteoporotic phenotype of ORX mice (33) whereas OT that can substitute for estrogen in OVX mice is unable to do so in male mice. This last discrepancy could be explained by the fact that OT and its receptor are regulated by estrogens and thus are located downstream in the estrogen signaling pathway. As a whole, these observations may lead to understand why OT can compensate for the estrogen defect in OVX regarding the bone phenotype whereas it is inefficient in the case of ORX mice that are lacking testosterone.

Androgens may not only impact muscle size or weight but also functional aspects such as muscle fiber type composition. In this way, testosterone treatment promotes lean mass as well as myofiber hypertrophy (34), and prevents the orchidectomy-induced shift of type I slow oxidative toward type IIa fast oxidative muscle fibers in slow muscle (35). Thus, testosterone regulates both the mass and function of skeletal muscle in adults. OT plasma levels and muscle stem cells OTR levels dramatically decline with age and demonstrate that OT is required for skeletal muscle tissue regeneration and homeostatic maintenance (19). In this recent work, Elabd and collaborators showed that acute OT treatment restored muscle regeneration in old mice, while OT signaling attenuation altered muscle regeneration in young mice (19). This effect seems to be triggered directly through muscle stem cell function. In other words, old mice deficient for OT displayed sarcopenia features. It is interesting to note that muscle mass declines with age in OT *knock-out* mice. The resistance of young mice could be related to high-testosterone levels, and the phenotype may be associated to the decrease in testosterone levels found in older mice (19). In our study, ORX mice displayed an expected loss of muscle mass, which was not rescued by OT treatment. This could be due to the interrelation between OT and testosterone pathways and can explain the inability of OT to rescue muscle mass decrease in ORX mice where testosterone level is very low.

In contrast to bone and muscle, OT treatment reversed orchidectomy-induced fat gain. It is accepted that hypogonadism induces adiposity in male, but the specific relation with visceral (intra-abdominal) adiposity is less documented compared to female mice (36). Indeed, visceral adiposity is a well-known risk factor for hypogonadism in male, due to the fact that this tissue is a key location for conversion of testosterone into estradiol, but the direct involvement of androgens on visceral fat development is not clear yet (37). Under physiological conditions, visceral ­adipose tissue mass is two times higher in male compared to female, independently of hypogonadism but correlated with physiological levels of estradiol (38). After menopause, and not andropause, this visceral fat mass increases dramatically (38). These data clearly link estrogens, and not androgens, to the development of this adipose tissue. OT treatment induces a decrease in subcutaneous and intra-abdominal adipose tissue of OVX and ORX mice, with a higher effect on intra-abdominal adipose tissue in female due to the fact that this tissue is specifically impacted after ovariectomy. Thus, it is tempting to hypothesize that this OT effect is mediated *via* an estrogen dependent pathway.

In conclusion, our present work associated with our previous published studies, demonstrated that OT is able to reverse features of hypogonadism directly related to estrogen pathway, but OT is not able to compensate the decline in androgen levels. OT seems to be an interesting alternative to estrogens treatment in post-menopausal women, and due to its important function in adiposity and in muscle regeneration, OT could be proposed in association with efficient testosterone treatment in men upon hypogonadism. Moreover, this kind of associated treatment could be very interesting in men displaying a defect in estrogen synthesis due to aromatase activity deficiency (39).

## Author Contributions

EA and DP designed the work. GB, MD, SB, and DP performed experiments. GB, CR, MS, DH, EA, and DP analyzed and interpreted data. All the authors were involved in manuscript writing and have approved the final version.

## Conflict of Interest Statement

The authors declare that the research was conducted in the absence of any commercial or financial relationships that could be construed as a potential conflict of interest.
